# The Nitrogen Availability Interferes with Mycorrhiza-Induced Resistance against *Botrytis cinerea* in Tomato

**DOI:** 10.3389/fmicb.2016.01598

**Published:** 2016-10-14

**Authors:** Paloma Sanchez-Bel, Pilar Troncho, Jordi Gamir, Maria J. Pozo, Gemma Camañes, Miguel Cerezo, Víctor Flors

**Affiliations:** ^1^Metabolic Integration and Cell Signaling Laboratory, Plant Physiology Section, Unidad Asociada al Consejo Superior de Investigaciones Científicas (Estación Experimental del Zaidín)-Department of Ciencias Agrarias y del Medio Natural, Universitat Jaume ICastellón, Spain; ^2^Department of Ciencias Agrarias y del Medio Natural, Universitat Jaume ICastellón, Spain; ^3^Department of Biology. University of FribourgFribourg, Switzerland; ^4^Department of Soil Microbiology and Symbiotic Systems, Estación Experimental del Zaidín, Spain Unidad Asociada-Department of Ciencias Agrarias y del Medio Natural, Universitat Jaume IGranada, Spain; ^5^Bioquímica y Biotecnología, Plant Physiology Section, Department of Ciencias Agrarias y del Medio Natural, Universitat Jaume ICastellón, Spain

**Keywords:** mycorrhiza-induced resistance, priming, tomato, *Botrytis cinerea*, nitrate tranceptors

## Abstract

Mycorrhizal plants are generally quite efficient in coping with environmental challenges. It has been shown that the symbiosis with arbuscular mycorrhizal fungi (AMF) can confer resistance against root and foliar pathogens, although the molecular mechanisms underlying such mycorrhiza-induced resistance (MIR) are poorly understood. Tomato plants colonized with the AMF *Rhizophagus irregularis* display enhanced resistance against the necrotrophic foliar pathogen *Botrytis cinerea*. Leaves from arbuscular mycorrhizal (AM) plants develop smaller necrotic lesions, mirrored also by a reduced levels of fungal biomass. A plethora of metabolic changes takes place in AMF colonized plants upon infection. Certain changes located in the oxylipin pathway indicate that several intermediaries are over-accumulated in the AM upon infection. AM plants react by accumulating higher levels of the vitamins folic acid and riboflavin, indolic derivatives and phenolic compounds such as ferulic acid and chlorogenic acid. Transcriptional analysis support the key role played by the LOX pathway in the shoots associated with MIR against *B. cinerea*. Interestingly, plants that have suffered a short period of nitrogen starvation appear to react by reprogramming their metabolic and genetic responses by prioritizing abiotic stress tolerance. Consequently, plants subjected to a transient nitrogen depletion become more susceptible to *B. cinerea*. Under these experimental conditions, MIR is severely affected although still functional. Many metabolic and transcriptional responses which are accumulated or activated by MIR such NRT2 transcript induction and OPDA and most Trp and indolic derivatives accumulation during MIR were repressed or reduced when tomato plants were depleted of N for 48 h prior infection. These results highlight the beneficial roles of AMF in crop protection by promoting induced resistance not only under optimal nutritional conditions but also buffering the susceptibility triggered by transient N depletion.

## Introduction

Beneficial microbe-plant associations are common in nature, and their benefits to plant health and their potential application in agriculture are under extensive scrutiny (Campos-Soriano and San Segundo, 2011; Smith and Smith, 2011). Among these associations, arbuscular mycorrhizal (AM) symbiosis is one of the most widespread mutualistic associations worldwide. AM symbioses are established between soil-borne fungi from the phylum Glomeromycota, known as arbuscular mycorrhizal fungi (AMF), and the roots of more than 80% of plant species. These fungi are ubiquitous obligate biotrophs that colonize plant roots to obtain plant carbohydrates for the formation, maintenance, and function of mycorrhizal structures and to complete their life cycle (Bago et al., 2000). In return, the AMF improve plant acquisition of water and mineral nutrients, as well as plants' ability to overcome biotic and abiotic stresses (Gianinazzi et al., 2010; Jung et al., 2012; Cameron et al., 2013; Pieterse et al., 2014; Selosse et al., 2014).

Multiple studies show that mycorrhizal plants are more resistant not only to root attackers but also to foliar pathogens and herbivorous insects (Campos-Soriano et al., 2011; Jung et al., 2012; Song et al., 2013, 2015). Although no antimicrobial compounds released by AMF have yet been described, it is known that AM plants can successfully resist root pathogens as well as certain foliar diseases. Among the mechanisms operating in the enhanced resistance, increased plant nutrition, competition for nutrients and colonization sites, and induction of defense mechanisms have been reported (Jung et al., 2012). In addition to these changes in the host plant, beneficial effects have been associated with changes in the microrhizosphere (Cameron et al., 2013). In fact, most reports suggest an induced resistance mechanism rather than increased tolerance or fungal competition between the AMF and the pathogen since AM plants are able to reduce the extent of the pathogen infection (Cordier et al., 1998; Campos-Soriano et al., 2011; Song et al., 2013; Hayek et al., 2014). This protection is known as mycorrhiza-induced resistance (MIR) (Pozo and Azcón-Aguilar, 2007; Jung et al., 2012) and in many respects resembles to rhizobacteria-induced systemic resistance (ISR) (Pieterse et al., 2014). The expression of defense genes in many of those studies was only reported during infection, hence following a defense priming pattern (Balmer et al., 2015), although direct activation of defenses has also been reported in mycorrhizal plants. For example, colonization of rice roots by AMF triggers signals that provide resistance against the blast fungus *Magnaporthe orizae* (Campos-Soriano et al., 2011). The mycorrhizal rice plants display resistance against *M. orizae* through a two-step mechanism: direct induction of defense genes in the absence of the pathogen and triggering defense priming once the infection is established. In this respect, it has been proposed that MIR acts through defense priming by pre-conditioning plant tissues for efficient defense activation (Pozo and Azcón-Aguilar, 2007; Jung et al., 2012). AMF can promote defense priming such that there are no fitness costs for the plant in terms of growth and yield depending on the developmental stages of the partners and the environmental conditions (e.g., nutrient availability and light intensity). In fact, there are reports describing the benefits in terms of fruit quality and plant fitness achieved mainly through improved uptake of phosphorous and nitrogen, although there are also reports showing a reduction in growth (Gianinazzi et al., 2010; Smith and Smith, 2011).

MIR has been associated with the metabolic and genetic rearrangement that occurs as a consequence of the AMF colonization of roots that affects plant primary and secondary metabolism (Rivero et al., 2015). The establishment and functioning of the symbiosis requires a high degree of coordination between both partners, and bidirectional (plant and fungal) control assures a fair trade of resources between the symbionts (Smith and Smith, 2011). Indeed, precise regulation of host hormone levels has been proposed as a central mechanism in the regulation of the interaction and could modulate plant defenses (Fernández et al., 2014; Gutjahr, 2014; Pozo et al., 2015). Several metabolic pathways have been shown to change following AMF root colonization, particularly phenol alcohols and oxylipin derivatives in tomato (Rivero et al., 2015). Seemingly, AMF colonization affects root gene expression and, remarkably, gene expression in shoots as well (Liu et al., 2007; Fiorilli et al., 2009; López-Ráez et al., 2010); metabolic changes in leaves have also been reported in mycorrhizal plants (Schweiger et al., 2014).

Although, significant progress has been made in recent years, the mechanisms by which AMF modulate plants' immune system are far from being understood. MIR may be a side effect of plants' mechanisms for regulating the level of fungal colonization in roots, which implies important changes in root hormone homeostasis (Pozo and Azcón-Aguilar, 2007; Pozo et al., 2015). During the first stages of colonization, mycorrhizal plants display a type of biotrophic pathogen response that involves regulation of SA responses (Güimil et al., 2005; Paszkowski, 2006; Jung et al., 2012). As mycorrhization progresses, there is a switch to an increase in JA-dependent responses. In fact, Rivero et al. (2015) demonstrated that most metabolites derived from α-linoleic acid, a precursor of JA, are induced in AMF-colonized roots.

Mycorrhizal associations and their benefits for plant health are affected by environmental conditions (Pozo et al., 2015). In particular, nutrient availability can have a strong impact on symbiosis and plant defenses (Pastor et al., 2014a,b). Despite the clear advantages acquired by mycorrhizal plants in the uptake of P, few studies have described the effect on N uptake and metabolism (Ames et al., 1983; Hawkins et al., 2000; Reynolds et al., 2005). Several ammonium (López-Pedrosa et al., 2006; Pérez-Tienda et al., 2011, 2012) and amino acid (Cappellazzo et al., 2008) transporters have been characterized in *Rhizophagus irregularis*. The sensing of nitrogen in the root environment has been a popular topic since it was demonstrated that nitrate transporters deliver signals to the shoot, regulating the C/N imbalance in the plant when there is an alteration of nitrogen in the root (Léran et al., 2015). In Arabidopsis, these signals contribute to plants' tendencies regarding growth or resistance to stress that is cross-regulated by the ABA repressor ABI2 and the nitrogen transporter NRT1.1. These dual functions of transport and signaling demonstrated by the membrane proteins were first described in animals, and the term transceptor was applied to them (Gojon et al., 2011).

The effect of nitrogen and its metabolism in plant immune defenses was extensively investigated by Fagard et al. (2014). A reduction in the N supply produces a decrease in chitinase, chitosanase and peroxidase activities, reducing Arabidopsis defenses (Dietrich et al., 2004). However, it is also known that high N fertilization may result in an increase in N-rich compounds, contributing to the activation of pathogenic virulence. In addition, the nature of the nitrogen supply is also a relevant factor in understanding how N affects immune responses. A continuous ammonium supply in tomato plants triggers *Pseudomonas syringae* resistance through systemic acclimation, activating basal defenses prior to infection (Fernández-Crespo et al., 2015). In fact, the high affinity ammonium transporter 2.3 (AMT2.3) is related to the suppression of premature arbuscule degeneration (PAD) in *Medicago truncatula* during the dynamic establishment and degeneration of the arbuscule process in root cells (Breuillin-Sessoms et al., 2015). Thus, it is clear that not only are the levels or sources of N important for AM establishment and the plant immune system, but the regulation and activity of nitrate and ammonium transporters are as well.

Four recent discoveries may suggest a relevant link between MIR and nitrate transporters. (1) The uptake of nitrate is a major issue in AM symbiosis (Fellbaum et al., 2012). (2) Changes in root architecture are a direct effect of AMF in the plant (García-Garrido and Ocampo, 2002). (3) In Arabidopsis, it was shown that nitrate transporters regulate the root architecture, particularly the *NRT2.1* gene that encodes a high-affinity transport system (Little et al., 2005). Finally, (4) the *Atnrt2.1* and *lin1* mutants, which are impaired in NRT2.1, display constitutive defense priming (Camañes et al., 2012; Gamir et al., 2014) because NRT2.1 acts as a repressor of plant immune responses against *Plectosphaerella cucumerina* and *P. syringae*.

In the present research, we aim to determine the effectiveness of MIR against *Botrytis cinerea* in tomato and the mechanisms behind it. We also aim to determine whether nitrogen sensing in the root environment and the consequent activation of nitrate transporters have an impact on the functionality of MIR. Here, we show that tomato plants colonized with *R. irregularis* display enhanced resistance against the aerial pathogen *B. cinerea*. AM plants show an enhanced horizontal defense that involves the potentiation of different defense pathways participating in basal resistance. Reasonably, transient nitrogen depletion induces NRT gene expression, which stimulates susceptibility. Despite this susceptibility, there are specific signals that still remain induced upon MIR. Those permanent changes may explain why AM plants are still more resistant against the pathogen even under low N conditions.

## Materials and methods

### Plant materials and mycorrhizal fungus inoculation

The AMF *Rhizophagus irregularis* (BEG 121) (formerly *Glomus intraradices*) was maintained as soil sand-based inoculum. Tomato seeds (*Solanum lycopersicum* L. cv. Better Boy) were surfaced-sterilized in commercial 10% HCl (v/v) and rinsed thoroughly with sterile water. The plants were germinated and maintained in a growth cabinet (16 h light-period and 26°C day and 18°C night) until transplant time and AMF inoculation. Subsequently, individual seedlings were transferred to 1 l pots with sterile vermiculite. Pots were inoculated by adding 10% (v:v) *R. irregularis* inoculum. The same amount of soil:sand mix but free of AMF was added to control plants. All control plants received an aliquot of a filtrate (<20 μm) of AM inocula to homogenize the microbial populations. For each treatment, a total of 10 plants were used. Plants were randomly distributed and grown in a greenhouse over a 16 h light period at temperatures of 26°C during the day and 18°C at night and at 70% humidity; the plants watered three times a week with Long Ashton nutrient solution (Hewitt, 1966) containing 25% of the standard phosphorus concentration. Plants were allowed to grow for four more weeks under greenhouse conditions before the nitrogen depletion treatments and pathogenic fungus inoculation. An aliquot of each individual root system was reserved for mycorrhizal quantification before subsequent experiments to ensure a good level of mycorrhization.

### Determination of mycorrhizal colonization

To determine the level of mycorrhization, soil debris was carefully removed and the roots were cut into 2 cm segments. Fungal structures within the roots were ink stained following the method described by Vierheilig et al. (1998). Roots were examined using a Nikon Eclipse 50i microscope under bright-field conditions. The percentage of total root length colonized by the mycorrhizal fungi was determined by the gridline intersection method (Giovannetti and Mosse, 1980).

### Nitrogen depletion experiments

The plants were maintained in a 25% in phosphorous Long Ashtom liquid solution with continuous aeration. A set of both non-mycorrhizal and mycorrhizal plants (AM) received a Long Ashton modified free-N solution for 48 h prior to pathogen inoculation.

### Inoculation with *botrytis cinerea* and trypan blue staining

Conidia of *Botrytis cinerea* CECT2100 (Spanish collection of type cultures, Universidad de Valencia, 46100 Burjassot, Spain) collected from 10- to 15-day-old PDA plates supplemented with tomato leaves at 40 mg ml^−1^ were pregerminated in Gambor's B5 medium (Duchefa, Haarlem, The Netherlands) supplemented with 10 mM sucrose and 10 mM KH_2_PO_4_ for 2 h in the dark with no shaking. Plant inoculation was performed on intact plants at 100% RH as described by Vicedo et al. (2009). Plants where inoculated by spraying a suspension of 1 × 10^6^ ml^−1^ spores of *B. cinerea* spores on the third and fourth leaves of the plant. Lactophenol Trypan Blue staining was performed on leaves infected to detect cell death (Saha et al., 1988).

### Total N measurements

Leave samples were dried at 65°C for 48 h. The samples were crushed in a hammer mill and weighed with a precision balance. The samples were analyzed for N total content using a TruSpec CHNS Micro elemental analyser (LECO Instruments S.L, Tres Cantos, Madrid).

### RNA extraction and RT-qPCR analysis

Gene expression by quantitative real-time RT-PCR was performed using RNA samples (3rd and 4th true leaves) extracted from leaf tissue using the RNA kit (Omega Bio-Tek Inc, Doraville, GA, USA) according to the manufacturer's instructions. To avoid contaminating DNA, the samples were treated with DNAse I. A total of 1 μg of total RNA was annealed to oligo-dT and reverse transcribed using an Omniscript Reverse Transcription kit (QIAGEN) to obtain cDNA. The sequences of the gene-specific oligonucleotides designed and used for real-time PCR are shown in Table S1. Real-time PCR was conducted using a QuantiTect™ SYBR Green PCR Kit (QIAGEN) and a StepOne instrument (Applied Biosystems). Serial dilutions of cDNA were used to create a standard curve to optimize amplification efficiency. All reactions were performed in triplicate. The specificity of RT-qPCR amplification was confirmed by the presence of a single peak in the melting temperature curve analysis. Relative quantification of specific mRNA levels was performed using the comparative 2^−Δ(ΔCt)^ method (Livak and Schmittgen, 2001). Expression values were normalized using the tomato elongation factor 1α (*SlEF1*α, acc. AB061263) as a housekeeping gene. Relative expression data were calculated from the difference in threshold cycle (ΔCt) between the studied genes and DNA amplified by primers specific for the *BcActin, NRT2.1, NRT2.2*; *NRT2.3*; *PROSYS, PR-1, JAR-1, LOX-D*; *ASR-1* and *PIN-II*.

For *B. cinerea* quantification, quantitative PCR was performed on plant extracts as described by Gamir et al. (2014), and fungal genomic DNA was extracted and quantified by comparing the expression of the housekeeping gene of the fungus *BcActin* as described by (Sanchez-Vallet et al., 2010).

### Liquid chromatography and ESI mass spectrometry

#### Targeted hormonal analyses

Leave samples stored at −80°C were freeze dried and powdered for subsequent analysis. Thirty milligrams of freeze dried powder was extracted at 4°C with 1 ml of H_2_O:MeOH (90:10) containing 0.001% of HCOOH and 100 ng/ml of internal standards. The samples were centrifuged at full speed for 15 min at 4°C. The supernatant was partitioned twice against diethylether. The fractions were pooled and dried in a speed vacuum and resuspended in H_2_O:MeOH (90:10) with 0.01% of HCOOH. A 20 μl aliquot was injected into an Acquity ultra-performance liquid chromatography system (UPLC) (Waters, Mildford, MA, USA), which was interfaced with a triple quadrupole mass spectrometer (TQD, Waters, Manchester, UK). LC separation was performed using an HPLC Kinetex C18 analytical column with a 5 μm particle size, 2.1 100 mm (Phenomenex). The chromatographic and mass spectrometry conditions were established as described by Gamir et al. (2012). The hormones 12-oxo-phytodienoic acid (OPDA), jasmonic acid (JA), JA-isoleucine (JA–Ile), abscisic acid (ABA) and salicylic acid (SA) were analyzed as described by Forcat et al. (2008).

#### LC-ESI full-scan mass spectrometry

Seventy-two hours following infection, the fourth and third true leaves were sampled and immediately frozen in liquid N_2_ and stored at −80°C until analysis. Thirty milligrams of freeze-dried leaf material was homogenized with 1 ml of MeOH:H_2_O (10:90) containing 0.01% of HCOOH at 4°C. Following centrifugation at full speed for 15 min at 4°C, the supernatant was filtered through 0.2 μm cellulose filters (Regenerated Cellulose Filter, 0.20 μm, 13 mm D. pk/100; Teknokroma). An aliquot of 20 μl was injected into an Acquity UPLC system (Waters, Mildford, MA, USA) interfaced with a hybrid quadrupole time-of-flight instrument (QTOF MS Premier). To accurately identify the signals detected, a second fragmentation function was introduced into the TOF analyser. This function was programmed in a t-wave ranging from 5 to 45 eV to obtain a fragmentation spectrum of each analyte (Agut et al., 2014; Gamir et al., 2014). Positive and negative electrospray signals were analyzed separately to obtain a global view of the data behavior. Analytes were eluted with a gradient of methanol and water containing 0.01% HCOOH. Three technical and three independent biological replicates per sample were randomly injected. The LC separation was performed using an HPLC SunFire C18 analytical column with a particle size of 5 μm, 2.1 × 100 mm (Waters). Solvent gradients and further chromatographic conditions were established as described by Gamir et al. (2014) and Agut et al. (2014).

To precisely identify metabolites, a library of plant metabolites was generated using chemical standards. The compounds from this library were characterized at the level of retention time, exact mass and spectrum fragmentation (Schymanski et al., 2014). Up to 84 compounds were prepared at a final concentration of 100 ppb in the same solution (Rivero et al., 2015). The standard solution was injected through the HPLC in both positive and negative ion mode for electrospray ionization (ESI) using the same conditions applied for the plant samples. For those compounds that were not represented in the internal library, the signals obtained in the non-targeted metabolomic analysis were confirmed by contrasting the fragmentation spectrum in the Massbank or the Metlin databases (www.massbank.jp; https://metlin.scripps.edu/).

#### Full-scan data analysis and bioinformatic processing

Data in the.raw format acquired with the Masslynx 4.1 software (Masslynx 4.1, Waters) were transformed into.cdf files using the Databridge tool. Chromatographic signals were processed using the software R for statistical purposes (http://www.r-project.org/). Signals from positive and negative electrospray ionization (ESI^+^; ESI^−^) analysis were processed separately. Peak peaking, grouping and signal corrections were developed by applying the XCMS algorithm (www.bioconductor.org; Smith et al., 2006). Metabolite amounts were analyzed based on the normalized peak area units relative to the dry weight. A non-parametric Kruskal-Wallis test (*p* < 0.05) was performed to test the metabolomic differences between treatments. Significance was assessed by the absence of overlapping in the box-and-plot graphical method. Principal component analysis and heat map analysis were carried out with the Mar-Vis Suit 2.0 software (Kaever et al., 2015), a tool for clustering and visualizing metabolic biomarkers. Adduct, isotope correction, clustering and color heat map visualization was also performed by using the packages MarVis Filter and MarVis Cluster. Data were combined for the PCA and heat map analysis but processed separately for compound identification and quantification.

#### Statistical analysis

Unless otherwise indicated in the figure captions, statistical analysis was carried out by one-way analysis of variance using the Statgraphics-plus software for Windows V.5 (Statistical Graphics Corp., MD, U.S.A.). Means were expressed by a standard error. They were compared using Fisher's least significant difference at 95%. All experiments were repeated at least three times. Each experiment involved a minimum of three plants.

## Results

### MIR is functional against *B. cinerea* in tomato but a transient N deficiency interferes with it, reducing the level of resistance

**Figure 1:**
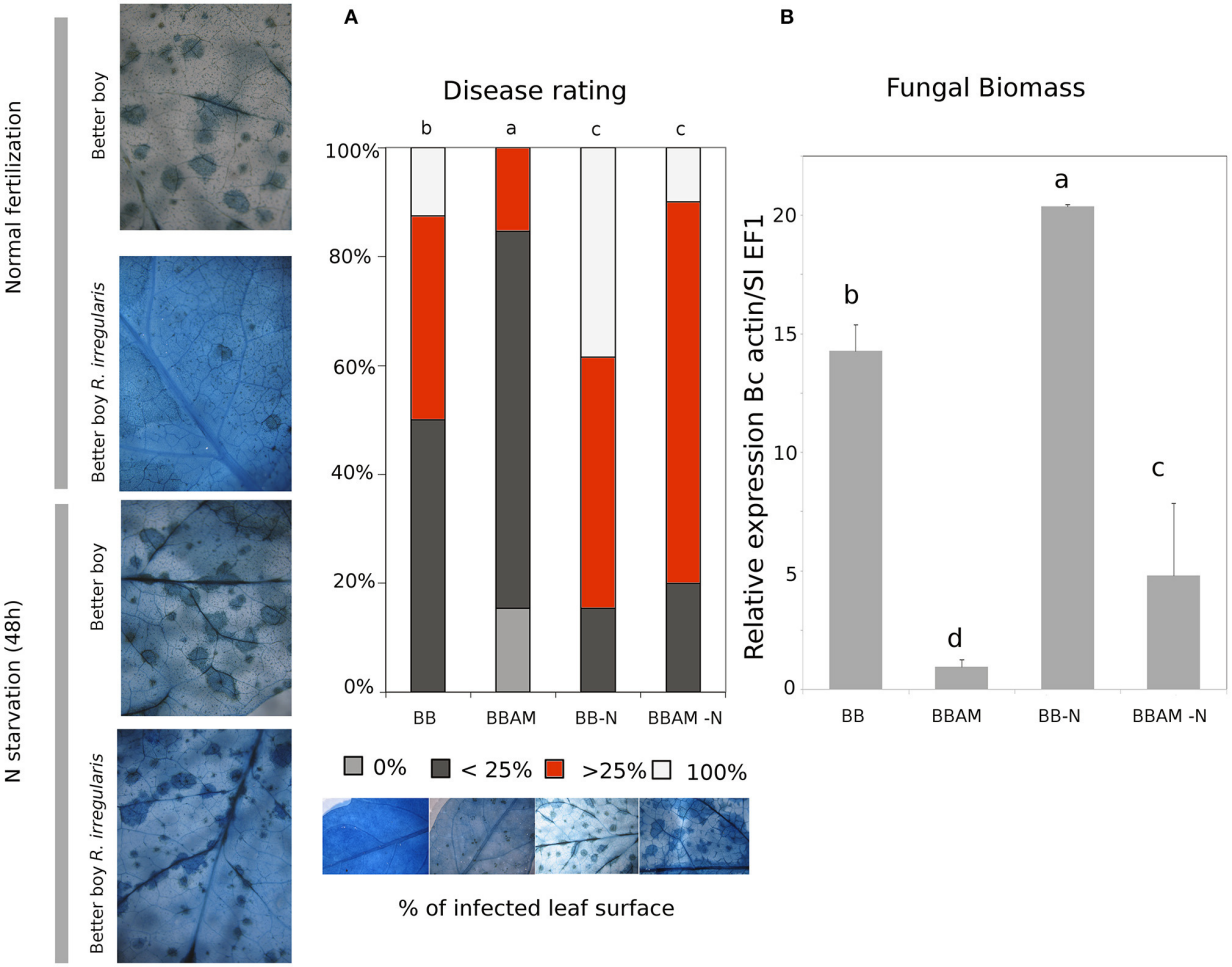
**Mycorrhiza-induced resistance and suppressive effect of a transient N depletion on MIR**. Images show trypan blue staining, where dark-blue spots are dead cells in *B. cinerea* infected plants. **(A)** The graphic shows the disease rating measured as a percentage of infected leaf surface and **(B)** fungal biomass measured as the ratio of relative expression of *Bcactin* gene referred to SlEF1α gene in non-mycorrhizal and AM plants under a normal fertilization regime or upon transient N starvation. BB, non-mycorrhizal Better Boy background; BBAM, Better Boy colonized by *R. irregularis*; −N, plants subjected to 48 h of nitrogen depletion; inf: plants infected with *B. cinerea*. Samples were harvested at 72 hpi. Different letters indicate statistically significant differences (ANOVA, Fisher's Least Significant Difference (LSD) test; *P* < 0.05, *n* = 3).

We first tested whether mycorrhiza-induced resistance (MIR) was functional in tomato cv. Better boy against the necrotrophic fungus *B. cinerea*. The assessment of cell death by trypan blue staining showed that mycorrhizal plants displayed significantly lower levels of damage (Figure 1A). This observation was confirmed by fungal biomass quantification by a comparative study between the housekeeping gene of the fungus *BcActin* and *SlEF1*α from the plant. Fungal biomass in the leaves was strongly reduced in plants colonized by *R. irregularis* (Figure 1B). Thus, MIR was functional against *B. cinerea* in tomato plants.

Previous studies indicated a complex regulation of nitrogen nutrition and nitrogen transporters (the so called transceptors) in the plant immune system (Gojon et al., 2011; Camañes et al., 2012; Fagard et al., 2014; Pastor et al., 2014b). To determine whether nutritional cues may interfere in MIR, plants grown with a full N supply were transferred to a modified solution in the total absence of N during 48 h. Subsequently, a set of plants were inoculated with the pathogen and compared with their respective controls (Figures 1A,B).

Notably, upon N starvation, non-mycorrhizal tomato plants became more susceptible compared with normally fertilized ones. Although, the disease rate assessed by trypan blue staining showed that N depletion fully abolished MIR (Figure 1A), the determination of fungal biomass showed that AM plants still were significantly more resistant than non-mycorrhizal plants (Figure 1B). Considering that trypan blue staining determines the rate of cell death, it is likely that the spread of fungal toxins beyond fungal mycelium may explain these apparent discrepancies between both determinations. Therefore, it is clear that N starvation increases plant susceptibility to the necrotrophic pathogen and partially impairs MIR although AM plants are still less susceptible to *B. cinerea* infection.

**Figure 2:**
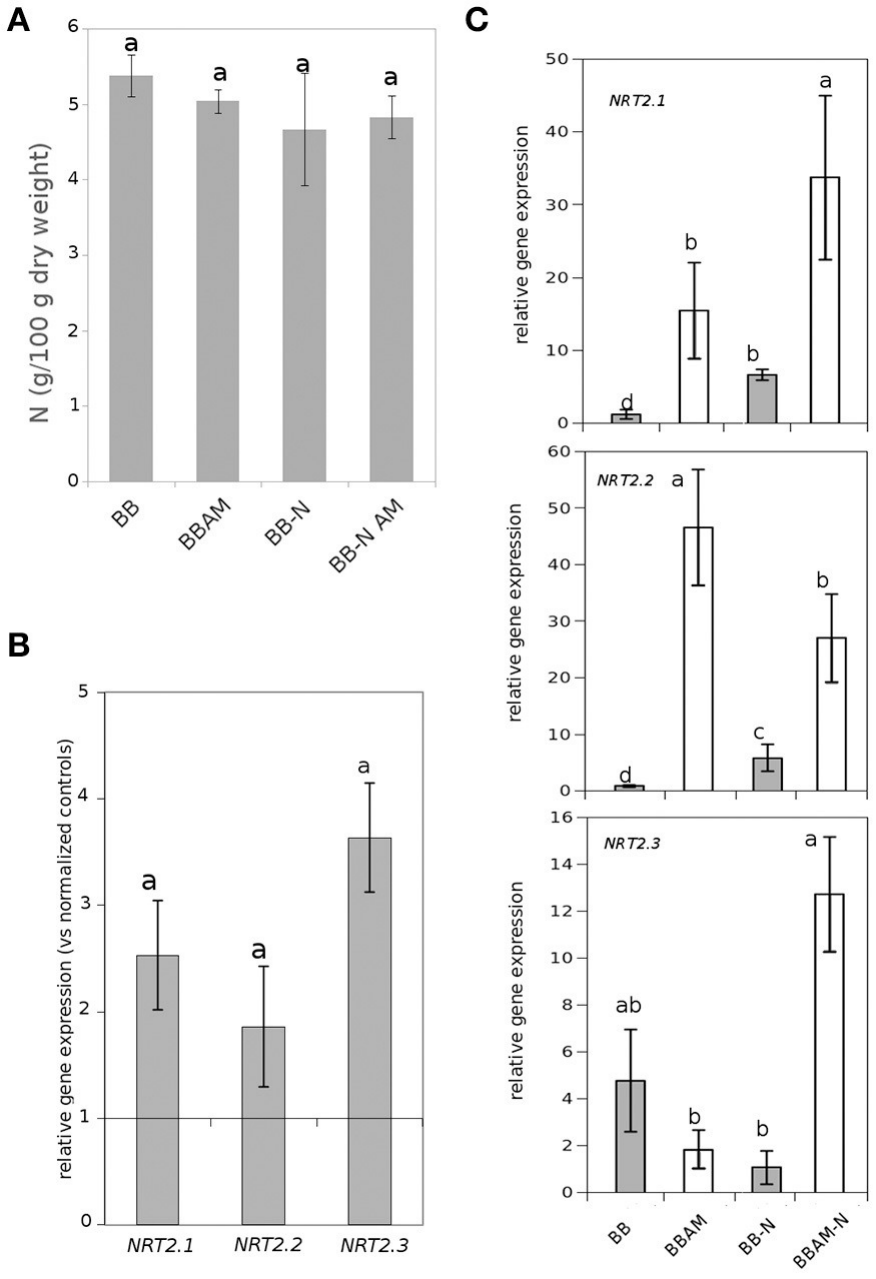
**Effect of N starvation on total N shoot levels and nitrate transporter gene expression**. **(A)** The nutritional status measured as N leaf content in non-infected plants and **(B)** the induction of nitrate transporters gene expression in leaves of non-mycorrhizal plants upon N starvation. Data are standardized with normally fertilized control plants. **(C)** Gene expression of nitrogen transporters in leaves of either non-mycorrhizal or mycorrhizal infected tomato plants at 72 hpi (BB, BBAM) normally fertilized or upon 48 h of nitrogen depletion (−N). Different letters indicate statistically significant differences (ANOVA, Fisher's Least Significant Difference (LSD) test; *P* < 0.05, *n* = 6).

We determined whether 2 days of N starvation was enough to alter the total N content in tomato leaves because the nutritional status influences plant susceptibility against fungal pathogens (Fagard et al., 2014). As shown in the Figure 2A, the total levels of N in the leaves did not differ significantly either in the AM plants or in those subjected to 48 h of N starvation. Three different nitrate transporters have been characterized in tomato, *NRT2.1, NRT2.2*, and *NRT2.3* (Hildebrandt et al., 2002; Fu et al., 2015). First, we observed that all three genes were upregulated in starved shoots, as previously shown in *Arabidopsis thaliana* for *NRT2.1* (Figure 2B; Camañes et al., 2012). In *B. cinerea* infected plants, *NRT2.1* and *NRT2.2* gene expression were upregulated by both mycorrhization and N starvation, showing a very similar profile of induction (Figure 2C), although it is clear that AM plants displayed much higher levels of expression than non-mycorrhizal ones. Surprisingly, the *NRT2.3* gene expression was rather different. Under standard N conditions, there were no significant changes in expression between pathogen-infected non-mycorrhizal or AM plants. However, N starvation only stimulated its expression in AM plants (Figure 2C). Thus, *NRT2.3* gene expression is only upregulated when both AMF and *B. cinerea* stimuli are present in the absence of N in the roots; otherwise, the gene expression is not significantly altered. These observations suggest a different functional regulation of NRT2.3 compared with the other two nitrate transporters.

### The untargeted metabolic profiling shows that MIR is a model of defense priming that is strongly altered by N depletion

**Figure 3:**
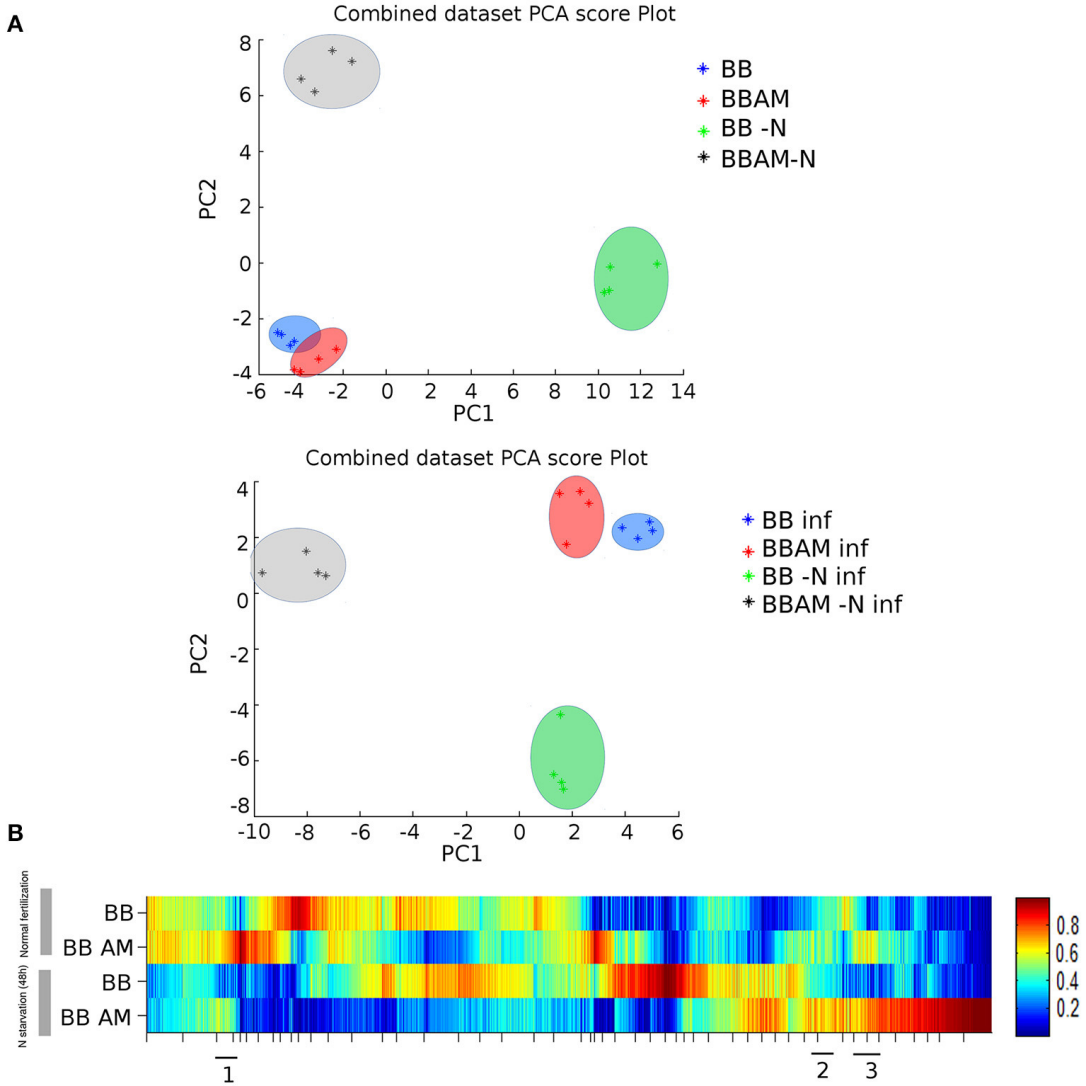
**Non-targeted metabolomic analysis of leave samples from infected non-mycorrhizal and mycorrhizal plants either normally fertilized or under a transient N depletion. (A)** Non-supervised principal component analysis (PCA) representation of the major sources of variability and **(B)** heat map analysis for combined ESI+ and ESI− signals obtained from a non-targeted analysis by HPLC-QTOF MS to monitor metabolomic changes during mycorrhization, 48 h of N starvation and fungal invasion at 72 hpi. Clusters 1, 2, and 3 of the heat map corresponding to metabolites accumulated in BBAM and BBAM-N plants upon infection were selected and analyzed in MarVis separately. (BB) Non-mycorrhizal tomato plants, (BBAM) mycorrhizal tomato plants, (−N) plants subjected to 48 h of nitrogen depletion, (INF) plants infected with *B. cinerea*. Leaf material from 3 individual plants was pooled for each treatment combination. Data points represent four independent experiments injected randomly into the HPLC-QTOF MS. Signals corresponding to different treatments were compared using the non-parametric Kruskal–Wallis test, and only data with a *P* < 0.01 between groups were used for subsequent processing.

To determine how AMF affects plants' metabolomic responses in the presence of infection and whether this metabolic fingerprint is altered upon transient N starvation, non-targeted metabolomic experiments were carried out. Both an unsupervised PCA and a heat map analysis were applied to the raw signals obtained from the Q-TOF mass spectrometer following biostatistics processing as described in the M&M section (Figures 3A,B). There was a clear separation between AM and control plants only under N starvation or pathogenic infection. According to component 1 (PC1), the two metabolic imprints were nearly the same, whereas they diverged when PC2 was observed. Interestingly, the effect of the infection induced a separation of the two treatments: Better Boy non mycorrhizal (BB) and Better Boy mycorrhizal plants (BBAM).

Unexpectedly, the transient depletion of N had a stronger effect on the separation of signals either in the presence or in the absence of the pathogen (Figure 3A). A similar conclusion was obtained from the heat map analysis of combined ESI^−^ and ESI^+^ (Figure 3B). There are abundant differences in the presence of infection between BB and BBAM plants, although the changes were much stronger when N was removed transiently from the nutrient solution (Figure 3B).

### Hormonal regulation of MIR against *B. cinerea* and N depletion impact

**Figure 4:**
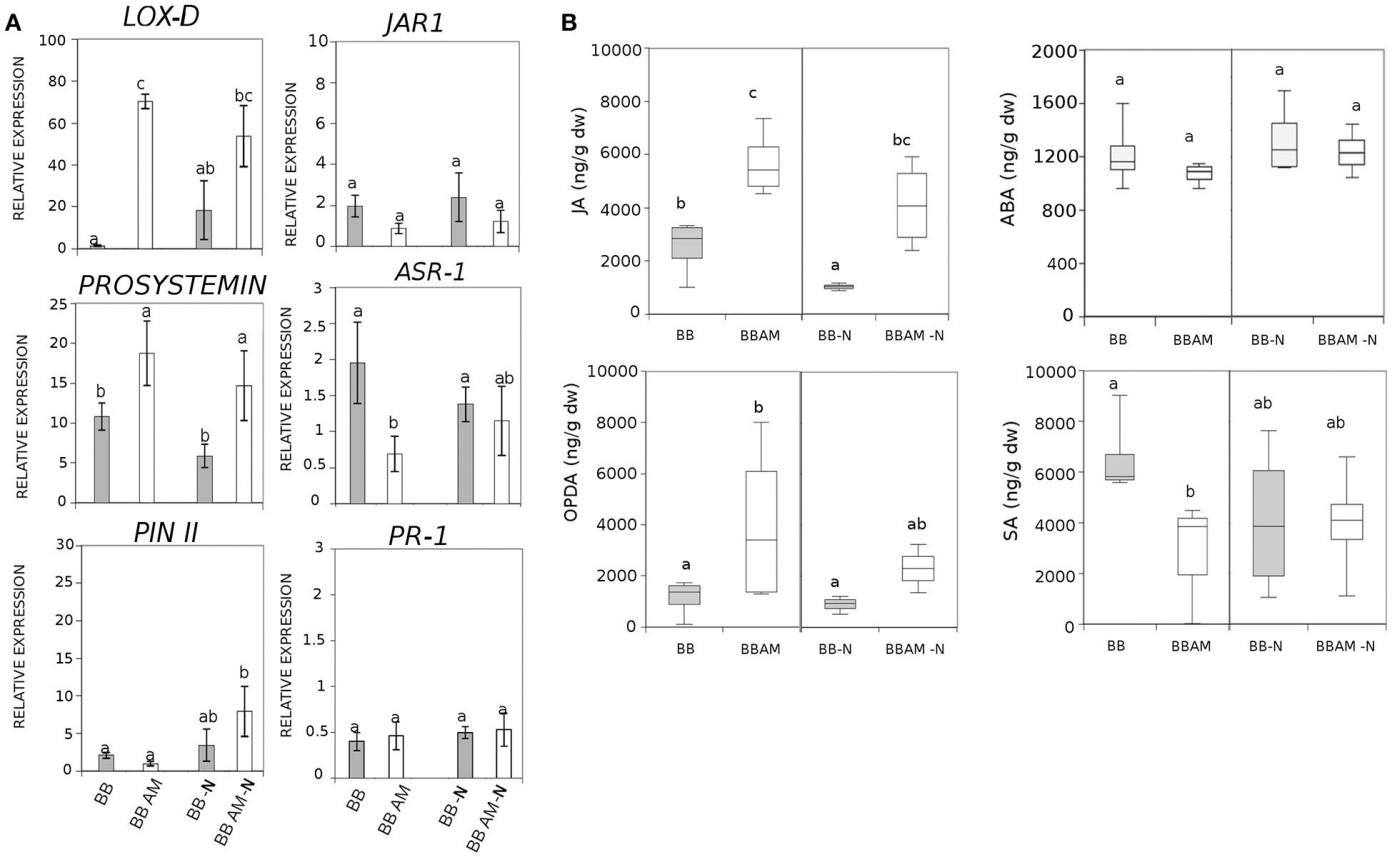
**Effect of N starvation in genetic and metabolic responses in the SA, JA, and ABA hormonal signaling pathways upon infection during MIR**. **(A)** Relative expression of marker genes of ABA, SA, and JA pathways. **(B)** Targeted analysis of hormones and levels of ABA, SA, JA, and OPDA in tomato plants. (BB) Non-mycorrhizal tomato plants, (BB AM) mycorrhizal tomato plants, (−N) plants subjected to 48 h of nitrogen depletion and fungal inoculation at 72 hpi. Leaf material from 3 individual plants at was pooled for each treatment combination. Different letters indicate statistically significant differences (ANOVA, Fisher's Least Significant Difference (LSD) test; *P* < 0.05, *n* = 6).

It is well known that SA, JA, and ABA, among other hormones, regulate plant immune responses and the induction of resistance (Pieterse et al., 2009, 2014). To determine whether MIR expressed in the leaves is under control of the main hormonal signaling pathways, we tested several marker genes and hormones within these pathways. Relative to the oxylipin pathway, the expression of *LOXD* and *PROSYSTEMIN* gene is clearly induced in infected mycorrhizal plants, although the N depletion does not have an effect on the expression of the genes. Surprisingly, other downstream marker genes such as *PINII* and *JAR1* do not change significantly upon N depletion or mycorrhization (Figure 4A). A similar situation was observed when ABA or SA marker genes were studied. The ABA marker *ASR1* gene was not induced by N depletion and was slightly reduced in infected mycorrhizal plants (Figure 4B). These observations were confirmed by the analysis of the corresponding hormones; JA and OPDA levels were both higher in mycorrhizal infected plants, whereas there was a clear reduction in SA levels, consistent with the negative JA-SA crosstalk.

Although, the N depletion abolished OPDA induction, JA still remained more accumulated in AM-N plants, which may explain why MIR is still functional under N deprivation. As expected, neither SA nor ABA changed significantly with N depletion regardless of the mycorrhizal status of the plant (Figure 4B). These results suggest a likely implication of the oxylipin pathway in MIR that is partially impaired upon N depletion.

### Analysis of the changes imposed by N depletion in the MIR phytometabolome profile

**Figure 5:**
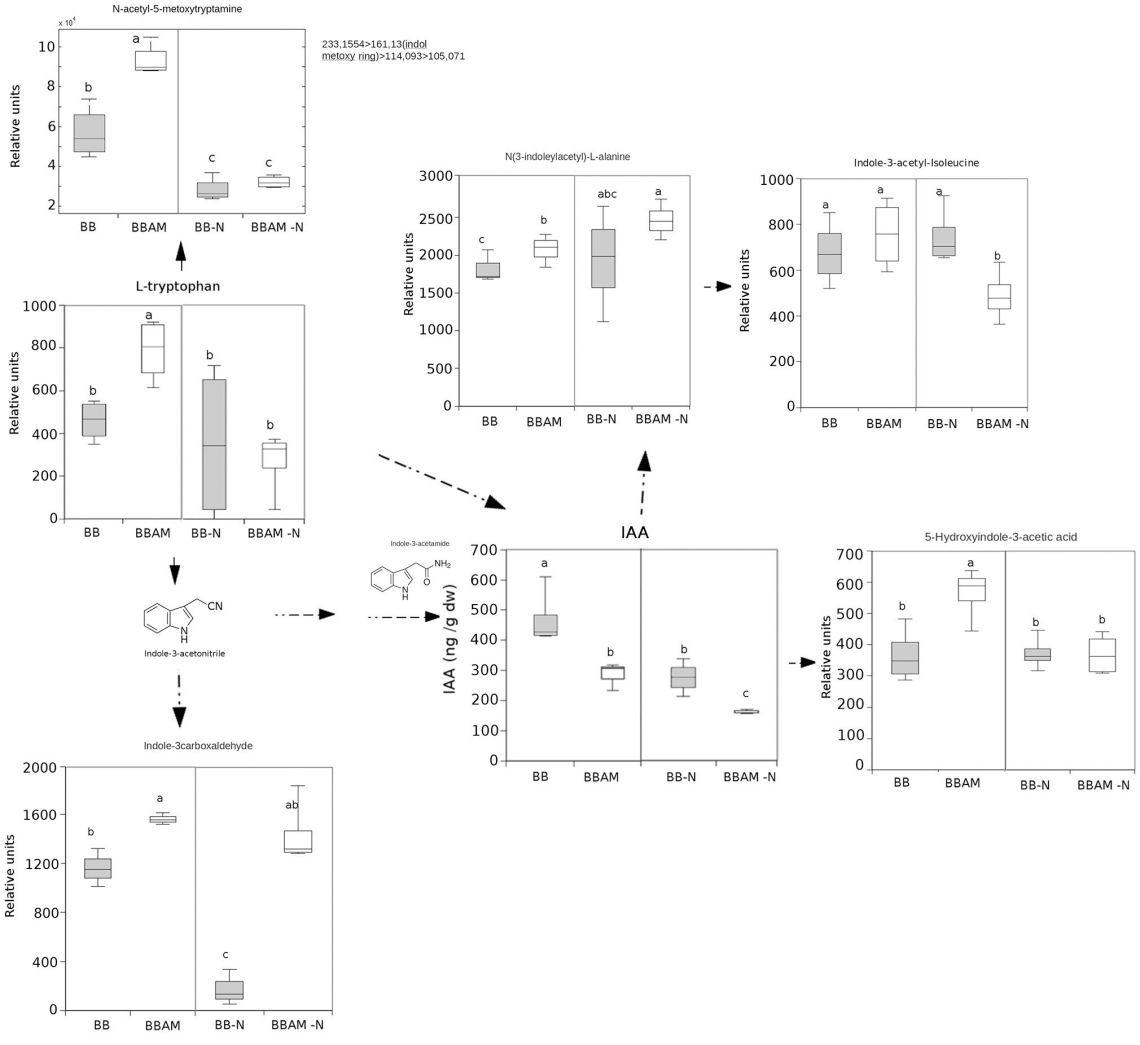
**Non-targeted profiling of indolic metabolites upon ***B. cinerea*** infection in non-mycorrhizal and mycorrhizal plants either normally fertilized or under a transient N depletion**. Non-mycorrhizal infected tomato plants (BB) and mycorrhizal infected tomato plants (BBAM) with (−N) or without 48 h of nitrogen depletion and fungal inoculation at 72 hpi were processed for relative quantification by HPLC-QTOF MS. The concentration of the metabolites was determined in all samples by normalizing the chromatographic area for each compound against the dry weight of the corresponding sample. All metabolites were precisely identified using chemical standards, except for N-acetyl-5metoxytriptamine, which was identified by its fragmentation spectrum. Leaf material from 3 individual plants was pooled for each treatment combination. Box plots represent the means for three independent experiments with two technical replicates. Dotted arrows indicate multiple metabolic steps; solid arrows indicate single steps. Different letters indicate statistically significant differences (ANOVA, Fisher's Least Significant Difference (LSD) test; *P* < 0.05, *n* = 6).

**Figure 6:**
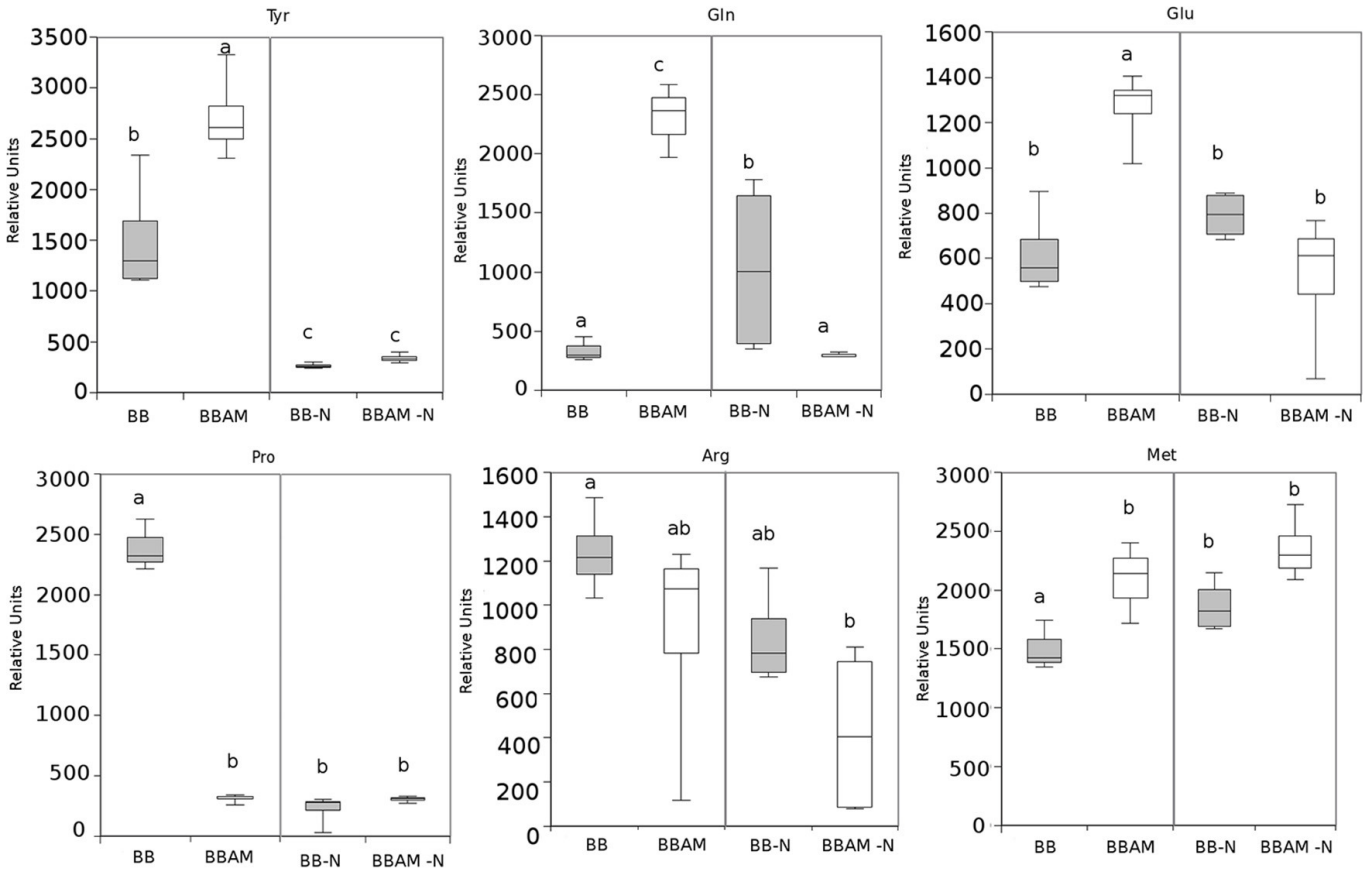
**Profiling of amino acids showing alterations upon ***B. cinerea*** infection in non-mycorrhizal and mycorrhizal plants either normally fertilized or under a transient N depletion**. Signal identity was confirmed by using chemical standards. Non-mycorrhizal infected tomato plants (BB) and mycorrhizal infected tomato plants (BBAM); (−N) plants exposed to 48 h of nitrogen depletion and fungal inoculation at 72 hpi. Leaf material from 3 individual plants was pooled for each treatment combination. Box plots represent the means for four independent experiments. Different letters indicate statistically significant differences (ANOVA, Fisher's Least Significant Difference (LSD) test; *P* < 0.05, *n* = 4).

**Figure 7:**
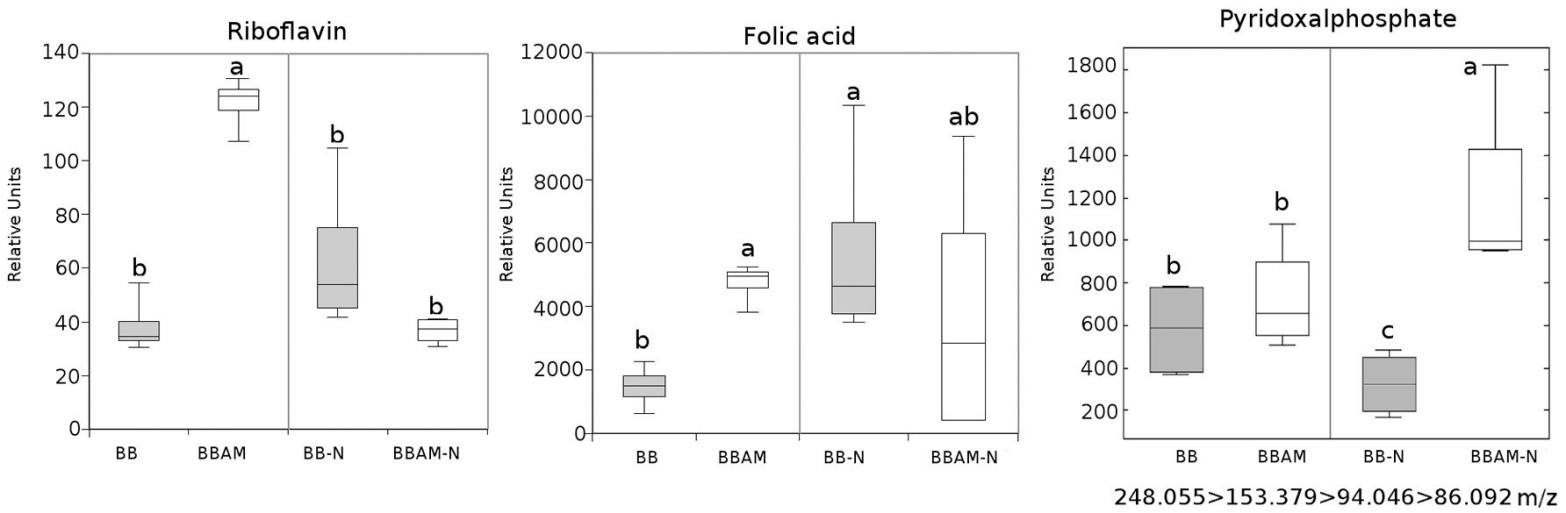
**Riboflavin, folic acid and pyridoxal phosphate profile upon ***B. cinerea*** infection in non-mycorrhizal and mycorrhizal plants either normally fertilized or under a transient N depletion**. Non-mycorrhizal infected tomato plants (BB) and mycorrhizal infected tomato plants (BBAM); (−N) plants exposed to 48 h of nitrogen depletion and fungal inoculation at 72 hpi. Riboflavin and folic acid were identified using chemical standards, whereas pyridoxal phosphate was identified using its fragmentation spectrum. Leaf material from 3 individual plants was pooled for each treatment combination. Box plots represent the means for three independent experiments with two technical replicates. Different letters indicate statistically significant differences (ANOVA, Fisher's Least Significant Difference (LSD) test; *P* < 0.05, *n* = 6).

By comparing the signals obtained in the non-targeted metabolomic analysis with an internal library of standards or by confirmation of the fragmentation spectrum in the databases, we could precisely identify many compounds that show enhanced accumulation during MIR against *B. cinerea*. In these groups of compounds, we found the amino acid Trp and its derivatives, such as N-acetyl metoxytriptamine, N(3-indoleylacetyl)-L-alanine, 5-hydroxyindole-3-acetic acid, and indole-3-carboxaldehyde (Figure 5). Among the analyzed amino acids, only Tyr, Gln, Glu, and Met were over-accumulated during MIR; however, except Met, the rest of the amino acids were reduced upon N depletion (Figure 6). Finally, the vitamins folic acid and riboflavin were also over-accumulated in MIR against *B. cinerea* (Figure 7). With the exception of indole-3-carboxaldehyde, the induction of all these metabolites during MIR was canceled in a nutrient-dependent manner and displayed the levels of non-mycorrhizal plants.

Thus, a transient N depletion abolished their accumulation in AM plants. Although these compounds are likely to contribute to the enhanced resistance against the fungus, they cannot be responsible for the enhanced resistance of mycorrhizal plants still functional upon transient N depletion because all of them display the same levels of non-mycorrhizal plants under nutrient stress conditions.

**Figure 8:**
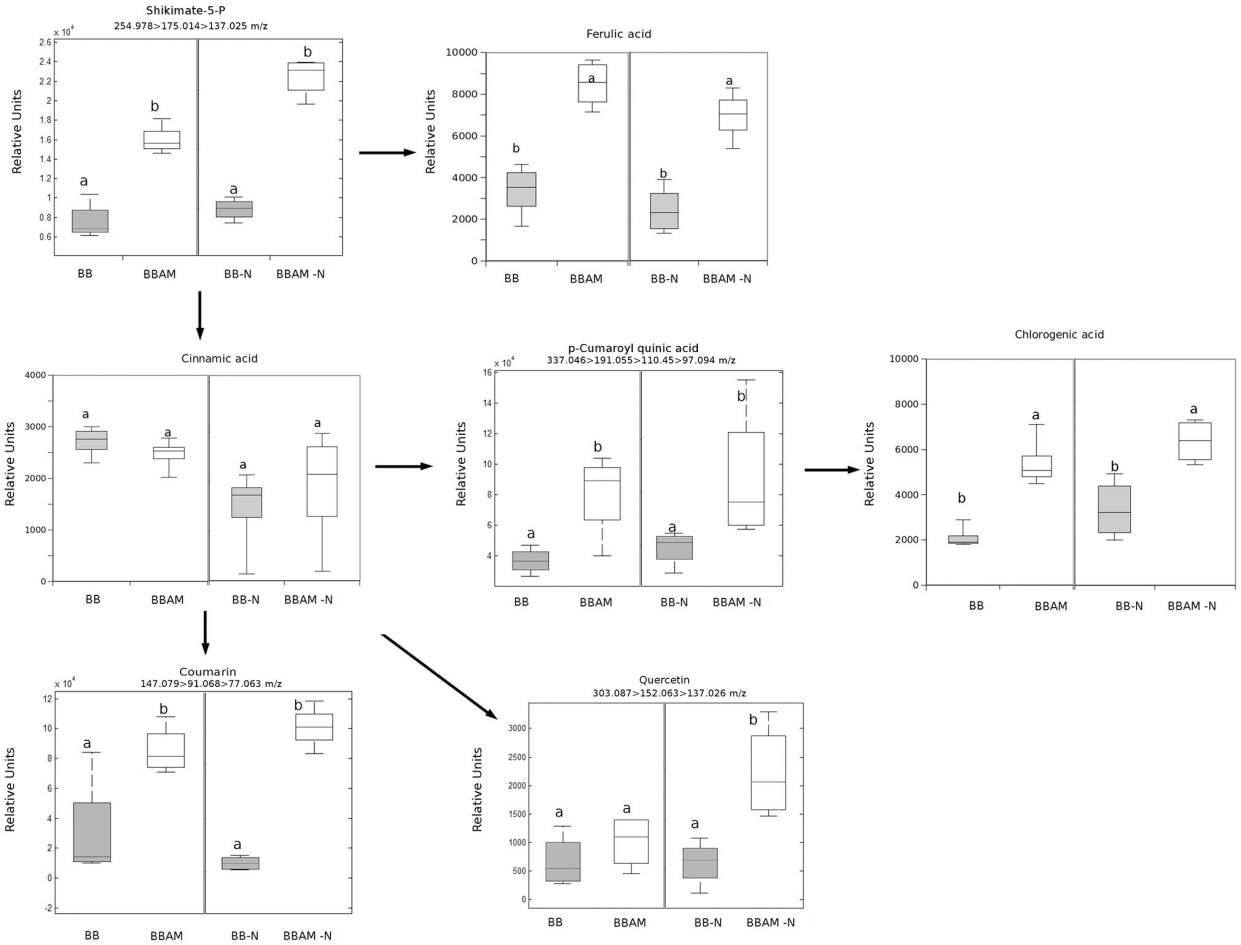
**Profile of phenolic acid and their derivatives upon ***B. cinerea*** infection in non-mycorrhizal and mycorrhizal plants either normally fertilized or under a transient N depletion**. Signal identity was confirmed by using chemical standards for chlorogenic acid and ferulic acid, and fragmentation spectra were used to identify the other phenols (see Table S2). Non-mycorrhizal infected tomato plants (BB) and mycorrhizal infected tomato plants (BBAM); (−N) plants exposed to 48 h of nitrogen depletion and fungal inoculation at 72 hpi. Leaf material from 3 individual plants was pooled for each treatment combination. Box plots represent the means for three independent experiments with two technical replicates. Different letters indicate statistically significant differences (ANOVA, Fisher's Least Significant Differences (LSD) test; *P* < 0.05, *n* = 6).

**Figure 9:**
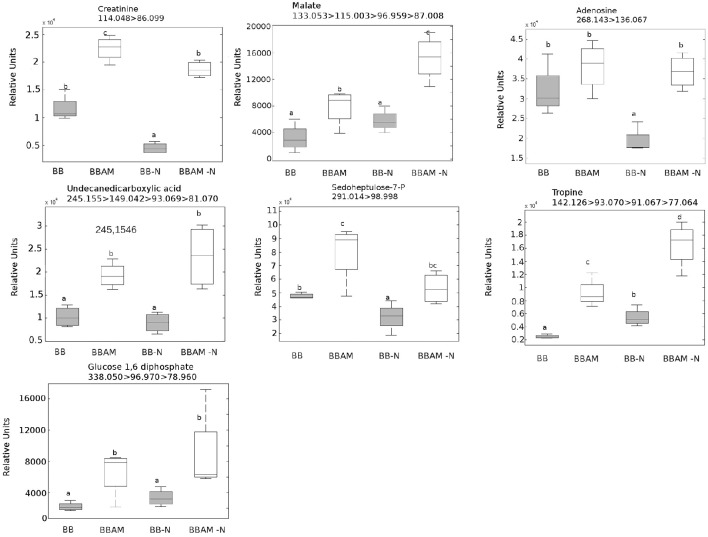
**Metabolites accumulated in mycorrhizal plants compared with non-mycorrhizal plants either normally fertilized or under a transient N depletion**. BBAM and BBAM-N plants belong to clusters 1, 2 and 3 of Figure 3. Metabolites were precisely identified by confirming their fragmentation mass spectrum. Non-mycorrhizal infected tomato plants (BB), mycorrhizal infected tomato plants (BBAM); (−N) 48 h of nitrogen starvation and fungal inoculation at 72 hpi. Leaf material from 3 individual plants was pooled for each treatment combination. Box plots represent the means for three independent experiments with two technical replicates. Different letters indicate statistically significant differences (ANOVA, LSD test; *P* < 0.05, *n* = 6).

Regarding to the metabolites that were accumulated in MIR under N stress, among all the compounds studied, only indole-3-carboxaldehyde and JA remained at higher levels in mycorrhizal plants upon N depletion. Therefore, the compounds may still be functional in promoting MIR under nutritional stress conditions. To determine what other signals potentially associated with MIR were still functional under the nutritional stress conditions, we selected clusters of compounds that were accumulated in mycorrhizal (BBAM and BBAM-N) compared with non-mycorrhizal (BB and BB-N) infected plants (Figure 4 clusters 1, 2 and 3). Surprisingly, in one of the clusters with the above-mentioned behavior, we observed an overrepresentation of phenolic acids and their derivatives (Figure 8). Shikimate-5-P, ferulic acid, p-coumaroyl quinic acid, chlorogenic acid, quercetin and coumarin levels were increased in BBAM-Ninf plants compared with the levels measured BB-Ninf plants. Several other compounds with the same response profile could be precisely identified by confirming their fragmentation mass spectrum (Figure 9). Among them, pyridoxal phosphate (Figure 7), malate (Figure 9) and tropine were even more strongly accumulated in BBAM-Ninf plants. Finally, a set of signals with the same profile could only be tentatively identified by exact mass (Figure S1). Notably, phenolics, malate and pyridoxal phosphate have been reported to mediate defense responses against pathogens and other stresses (Pastor et al., 2014a; Li et al., 2016). Therefore, these metabolites are likely to contribute to the functionality of MIR under nutritional stress conditions.

## Discussion

Mycorrhiza-induced resistance has been shown to be functional against different biotic stressors such as pathogens and insects (Jung et al., 2012; Cameron et al., 2013; Smith and Smith, 2015). AMF confers enhanced resistance not only against root infections but also against leaf pathogens. Reports on MIR suggest that this induced resistance occurs through a defense priming mechanism (Campos-Soriano et al., 2012; Song et al., 2015). In tomato, symbiosis with the AMF *Funneliformis mosseae* has been shown to reduce early blight promoting oxylipin-related gene expression and primed induction of glucanase, chitinase, PAL, and LOX enzymatic activities upon *Alternaria solani* infection (Song et al., 2015). *F. mosseae* has also been shown to protect tomato against *B. cinerea*; although, no molecular mechanisms have been proposed for such protection (Fiorilli et al., 2011).

In the present study, we provide evidence of the ability of the widely distributed AMF *R. irregularis* to protect tomato plants against the foliar necrotrophic pathogen *B. cinerea*. Furthermore, we also found that this protection occurs through defense priming. As indicated by the PCA of the metabolic profiles obtained by untargeted analysis, however, a certain effect in the leaf metabolome is associated with mycorrhization in the absence of challenge. The changes were particularly pronounced when plants were under pathogen attack or perceiving nutritional depletion.

Indeed, quantification of the fungal biomass showed that MIR is still functional under nutritional stress, although to a lesser extent than in plants under normal N conditions. As expected, tomato plants reorganize with transcriptional and metabolic profiles upon infection. First, the JA-related genes *LOXD* and *PROSYS* are significantly induced during MIR. Systemin was shown to be an activator of the oxylipin pathway (Farrokhi et al., 2008), acting through a positive feedback loop with JA biosynthesis (Ryan and Pearce, 1998). In agreement with this enhanced activation of JA biosynthetic genes, levels of JA and its precursor OPDA are higher in infected mycorrhizal plants. In contrast, the finding that leaves from mycorrhizal infected plants show a significant reduction in SA levels can be explained by the negative interference of the elevated levels of JA (Flors et al., 2008). Remarkably, it has been shown that in tomato, enhanced SA levels benefits *B. cinerea* infection by antagonizing the effective JA-dependent defenses (El Oirdi et al., 2011). Our data suggest that this manipulation by the pathogen is somehow avoided in mycorrhizal plants. As part of the complex defense priming triggered by AMF, the indolic compounds may play a relevant role. Trp, Indole carboxadehyde, N-acetyl-5 metoxytriptamine and 5-hydroxy-3-acetic acid were over-accumulated in infected AM tomato compared with their accumulation levels in non-mycorrhizal plants. Notably, Met, Tyr, Gln, and Glu were also induced in BBAM-infected plants. Vitamins such as riboflavin and pyridoxal phosphate (PLP) have been also related to defense priming in Arabidopsis against *B. cinerea* and other pathogens (Zhang et al., 2009, 2014, 2015). Remarkably, the N required for PLP biosynthesis appears to be supplied by the hydrolysis of GLn to Glu through the salvage biosynthetic pathway of vitamin B6 (Zhang et al., 2014); thus, elevated Glu and PLP levels may be related. Glu participates in signal transduction from systemic tissues upon wounding (Christmann and Grill, 2013). Whether Glu is translocated systemically from the roots to the infected shoots still remains to be determined, although recently Rivero et al. (2015) showed that Glu is strongly accumulated in AM roots; however, it is noteworthy that Glu has also been associated with other defense priming inducers in Arabidopsis, such as BABA or infection with avirulent bacteria (Pastor et al., 2014a). In addition, one of the most representative pathways among the hits in the over-accumulated signals of infected AM plants was the phenolic acid derivatives pathway. Again, this pathway was induced in *R. irregularis* colonized tomato roots, showing enhanced accumulation of the lignans precursors and shikimate derivatives (Rivero et al., 2015). The potential contribution of the metabolite changes in the roots to the observed alterations in shoots deserves further investigation.

As a partial conclusion, it is worth noting that all these metabolic and genetic changes are likely to contribute to the resistant phenotype of AM tomato plants because most of them have been previously linked to plant resistance against pathogens.

Surprisingly, most of these signals are repressed when the plant senses nutritional stress. In Arabidopsis, N starvation on one side of the root system leads to an up-regulation of nitrate uptake on the other side of the root system accompanied by an up-regulation of the nitrogen transporter NRT2.1. (Gansel et al., 2001). The closest sequence homologies to the Arabidopsis *NRT2.1* are tomato *NRT2.4* with 70% homology and *NRT2.3* with 69% homology, both encoding functional transporters (Fu et al., 2015). Remarkably, both genes are expressed in leaves upon N starvation, suggesting additional functions in N uptake in the roots.

Plants subjected to 48 h of N depletion induce *NRT2.1* gene expression, as described previously for other *NRT* family members in Arabidopsis (Camañes et al., 2012; Kiba et al., 2012). There is increasing evidence that N supply modulates not only primary plant responses such as root architecture and C/N metabolism (Lejay et al., 1999; Remans et al., 2006; Feng et al., 2011) but also plant immune responses (Camañes et al., 2012; Fagard et al., 2014; Pastor et al., 2014b; Fernández-Crespo et al., 2015). In the present study, we showed that roots' perception of nitrogen starvation enhanced *B. cinerea* susceptibility and reduced but did not impair MIR. The experimental conditions we implemented prevented N scarcity in the plant because we only exposed adult tomato plants to a depletion period of 48 h, which is not sufficiently long to alter levels of total N in the leaves. Despite this observation, the transient depletion has a strong effect on the plant metabolome as it is observed in the unsupervised PCA analysis. This finding demonstrates that the sensing of N depletion induces a metabolic rearrangement preparing the plant for likely N starvation. Previous studies have shown that lower N regime levels and transient N starvation increase the susceptibility of Arabidopsis to *A. alternata* and *P. cucumerina* (Camañes et al., 2012; Pastor et al., 2014b). Furthermore, Vega et al. (2015) showed that tomato cv Micro-Tom is more susceptible to *B. cinerea* under low nitrogen fertilization regimes (<4 mM KNO_3_). This reduction of N has a strong effect on primary and secondary metabolism gene transcripts and appears to be directly linked to oxidative stress. These studies propose a reorganization of plants' metabolism to address N starvation, which interferes with the plant immune system, transforming the plant into a more susceptible host for the pathogens. In our study, we showed that although MIR effectiveness is severely affected by transient N depletion, it is still functional, allowing for a reduction in pathogen proliferation in the plant tissues. This finding demonstrates the relevance of using AMF in field crops, which may preserve a better status of plants' immune system during nutritional fluctuations.

The strong effect of N depletion, likely mediated by *NRT2* transcript induction, was the abolishment of the accumulation of OPDA, most Trp and indolic derivatives, riboflavin, folic acid and amino acids upon infection compared with levels measured in BBAM plants not subjected to N starvation prior to infection. These compounds have been previously shown to participate in defense against *P. cucumerina*, as is the case for Trp derivatives (Bednarek et al., 2009; Gamir et al., 2014), against *P. syringae* in the case of riboflavin (Zhang et al., 2009) and against *B. cinerea* in the case of Glu (Hatmi et al., 2015). Our observations indicate that all members of the *NRT2* family are induced upon N depletion without infection. It is likely that in tomato these members function as transceptors, as demonstrated in Arabidopsis for *NRT2* family members (Gojon et al., 2011; Camañes et al., 2012). In Arabidopsis, the de-repression of *NRT2.1* triggers susceptibility by repressing defenses against *P. syringae* and *P. cucumerina* (Camañes et al., 2012; Gamir et al., 2014). Notably, *lin1* Arabidopsis mutants, blocked in *NRT2.1*, express constitutive defense priming and are more resistant to necrotroph pathogens. Our results show that *NRT2.1* and *NRT2.2* are induced in mycorrhizal plants but that *NRT2.3* is repressed. Considering that *NRT2.3* shows high homology with Arabidopsis *NRT2.1*, it is likely that AMF modulates its induction upon *B. cinerea* infection, and subsequently, the plant becomes more resistant. Further research is required to understand how *NRT2* members regulate plant immune responses in tomato. However, the fact that under N depletion all three members become strongly induced in infected plants and MIR is less effective confirms the hypothesis that *NRT2.3* may function as a sensor of nutritional stress repressing immune defense responses in the plant. Clearly, *NRT2.3* induction and the repression of all the metabolic signals described above upon transient N deprivation simply block portion of the primed defenses induced in mycorrhizal plants. Indeed, there is a significant part of the phytometabolome that still remains strongly induced, and therefore, these compounds are enhanced in a N-independent manner. The indole-3-carboxaldehyde is of particular interest. Although its role in defense responses is not currently known, its oxidized form, indole-3-carboxylic acid, was recently described as a signal found in a common fingerprint in several priming stimuli (Gamir et al., 2014). There are many hits in the phenolic acid pathway and derivatives accumulated in AM-N-infected plants. Previous reports describe the antifungal and defensive function of many of these compounds, particularly the flavonoid quercetin and chlorogenic acid (Jia et al., 2010). The protective roles of malic acid and vitamin B6, mainly on its vitamer piridoxal phosphate, are also well known (Zhang et al., 2014). Both compounds have been described to participate in induced defense and priming mechanisms (Pastor et al., 2014a; Zhang et al., 2014 respectively). How sedohetulose-7-phosphate, creatine, glucose1,6-diphosphate, adenosine or tropine, and other partially identified compounds with similar accumulation patterns mediate MIR either in normally fertilized plants (BBAMinf) or under N depletion (BBAM-Ninf) remains unknown at the moment.

In conclusion, we have shown that *R. irregularis* colonization of tomato roots results in an increased resistance of the shoots to *B. cinerea*, likely through defense priming involving the induction of a set of secondary metabolites, including the oxyilipins JA and OPDA; Trp derivatives; the amino acids Tyr, Met, Gln, and Glu; some indoles; and phenolic acids and their derivatives. All of these metabolites appear to participate as an integral part of a complex tuning mechanism of the immune system in mycorrhizal plants. However, plants that sense potential N starvation (transient N depletion in our experimental system) reorganize their metabolism to prepare for a nutritional battle antagonizing defense responses against biotic stress. Interestingly, this defense repression observed in non-mycorrhizal plants is partially antagonized in *R. irregularis* colonized plants that maintain an active part of the N-independent phytometabolome changes related to resistance, mounting a less effective but still functional MIR against *B. cinerea*.

## Author contributions

PS: Genetic análisis, mycorrhization expériments, hormonal análisis. PT: mycorrhization assays, fungal inoculation. JG: metabolomic analysis. MP: Mycorrhization % determination, microscopy. GC: nitrogen content analysis. MC: NRT gene expression. VF: data analysis and processing, bioinformatic analysis of the metabolimic data, figure elaboration, manuscript writing, coordination.

### Conflict of interest statement

The authors declare that the research was conducted in the absence of any commercial or financial relationships that could be construed as a potential conflict of interest. The reviewer and handling Editor declared their shared affiliation, and the handling Editor states that the process nevertheless met the standards of a fair and objective review.

## Supplementary material

Figure S1 **Metabolites accumulated in AM and AM-N plants corresponding to clusters 1, 2, and 3 of Figure 3 tentatively identified by exact mass**. Non-mycorrhizal infected tomato plants (BB), mycorrhizal infected tomato plants (BBAM); (−N) 48 h of nitrogen starvation and fungal inoculation at 72 hpi. Leaf material from 3 individual plants was pooled for each treatment combination. Box plots represent the means for three independent experiments with two technical replicates. Different letters indicate statistically significant differences (ANOVA, LSD test; *P* < 0.05, *n* = 6).

Table S1 **Primers used for qPCR analysis**.

Table S2 **Daughter ions of phenolic compounds obtained following fragmentation in a T-Wave analyzer using a collision energy that ranged from 5 up to 45 eV**.
