# Supplementary figures and images for: The Nitrogen Availability Interferes with Mycorrhiza-Induced Resistance against Botrytis cinerea in Tomato
Source: Front Microbiol. 2016 Oct 14;7:1598. doi: 10.3389/fmicb.2016.01598 (PMC5064179; doi:10.3389/fmicb.2016.01598)

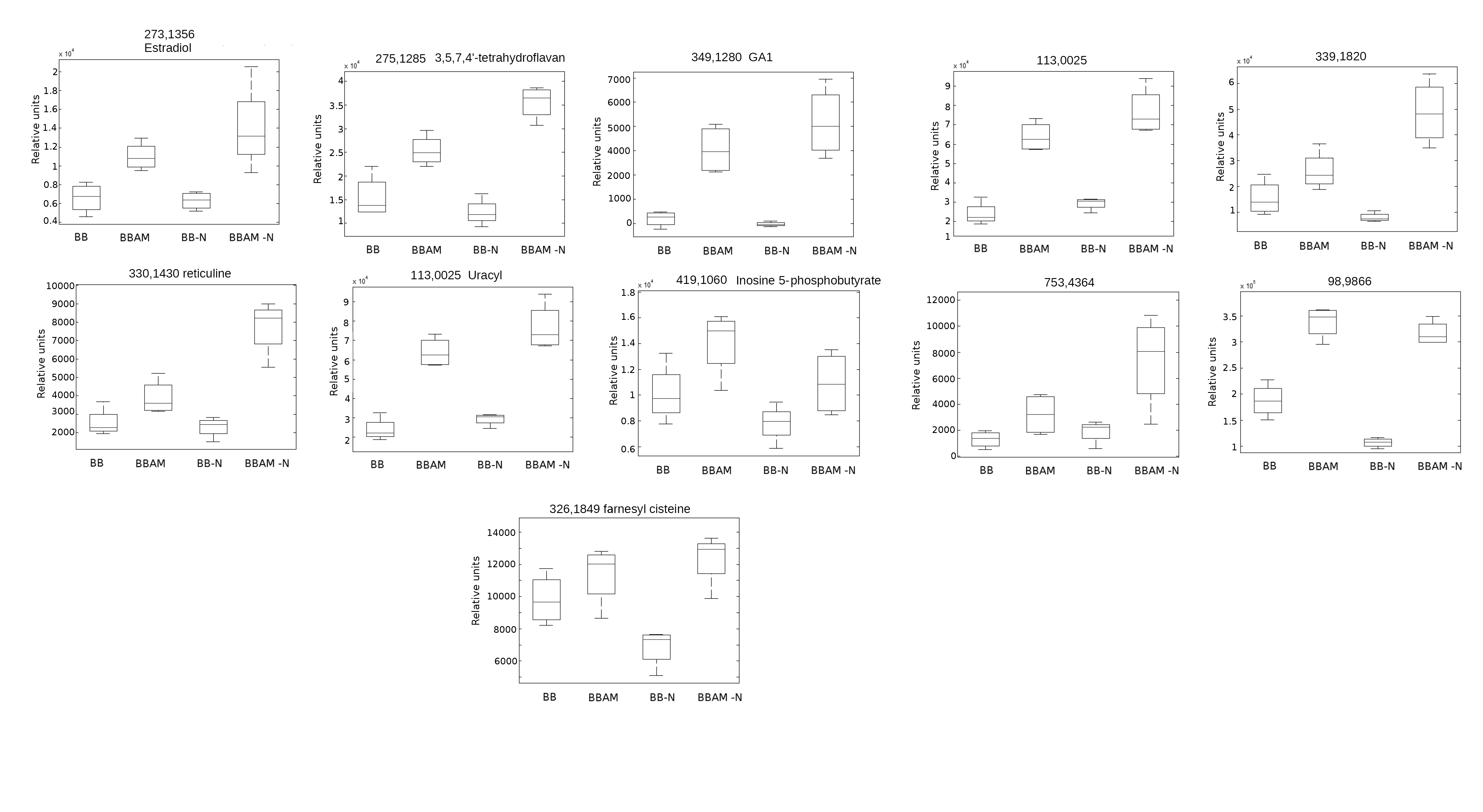

Supplement: Figure S1 — Metabolites accumulated in AM and AM-N plants corresponding to clusters 1, 2, and 3 of Figure 3 tentatively identified by exact mass. Non-mycorrhizal infected tomato plants (BB), mycorrhizal infected tomato plants (BBAM); (−N) 48 h of nitrogen starvation and fungal inoculation at 72 hpi. Leaf material from 3 individual plants was pooled for each treatment combination. Box plots represent the means for three independent experiments with two technical replicates. Different letters indicate statistically significant differences (ANOVA, LSD test; P < 0.05, n = 6). [file Image1.JPEG]
