# Supplementary material for: The Nitrogen Availability Interferes with Mycorrhiza-Induced Resistance against Botrytis cinerea in Tomato
Source: Front Microbiol. 2016 Oct 14;7:1598. doi: 10.3389/fmicb.2016.01598 (PMC5064179; doi:10.3389/fmicb.2016.01598)
Supplement: Table S1 — Primers used for qPCR analysis. [file Table1.DOC]

Supplementary table 1. Primers used for qPCR analysis.

| LOX-D | GACTGGTCCAAGTTCACGATCC----ATGTGCTGCCAATATAAATGGTTCC |
| --- | --- |
| PIN-II | GAAAATCGTTAATTTATCCCAC---ACATACAAACTTTCCATCTTTA |
| EF1 | GATTGGTGGTATTGGAACTGTC---AGCTTCGTGGTGCATCTC |
| PR-1 | ATGTGTGTGTTGGGGTTGGT---ACTTTGGCACATCCAAGACG |
| PROSYSTEMIN | AATTTGTCTCCCGTTAGA----AGCCAAAAGAAAGGAAGCAAT |
| Bc-TUB | CCGTCATGTCCGGTGTTACCAC---CGACCGTTACGGAAATCGGAA |
| JAR-1 | CAT TGA AAC CAT CTC CTT GA---TAA ACT GCT TGC TGC TGT AAA |
| ASR-1 | ACA CCA CCA CCA CCA CCT GT---CTG TTT GTG TGC ATG TTG TTGA |
| NRT2.1 | TTC CTG TTA CAT TTT GTC ATT TCCC---CAG ATT CAA GAC TAT CCA TTC CTC |
| NRT2.2 | TCA AGG GAA CGG AAG AAC ATT ATTA---GCT CAT TGA ACT AAA GAT TGA CGA T |
| NRt2.3 | AAT GCA TGG TGT TAC TGG TAG AGAG---CTA ATA ATA GGG ACT AAA GGG GCTA |
