# Supplementary material for: The Nitrogen Availability Interferes with Mycorrhiza-Induced Resistance against Botrytis cinerea in Tomato
Source: Front Microbiol. 2016 Oct 14;7:1598. doi: 10.3389/fmicb.2016.01598 (PMC5064179; doi:10.3389/fmicb.2016.01598)
Supplement: Table S2 — Daughter ions of phenolic compounds obtained following fragmentation in a T-Wave analyzer using a collision energy that ranged from 5 up to 45 eV. [file Table2.DOC]

**Table S2. Daughter ions of phenolic compounds obtained following fragmentation in a T-Wave analyzer using a collision energy that ranged from 5 up to 45 eV.**

| **Compund** | **Electro-spray mode** | **Transition m/z** | **Marker** |
| --- | --- | --- | --- |
| p-Cumaroyl quinic acid | ESI- | 337.046>191.055>110.45>97.094 | 72 |
| Quercetin | ESI- | 303.087>152.063>137.026 | 266 |
| Shikimate | ESI- | 254.978>175.014>137.025 | 93 |
| Cuomarin | ESI- | 147.079>91.068>77.063 | 448 |
